# 74957 Utilizing 3D Printing to Assist Planning of Percutaneous/Endovascular Procedures in Interventional Radiology

**DOI:** 10.1017/cts.2021.564

**Published:** 2021-03-30

**Authors:** Lucas Richards, Shiv Dalla, Carissa Walter, Aaron Rohr

**Affiliations:** University of Kansas Medical Center

## Abstract

ABSTRACT IMPACT: We plan to measure the impact of integrating 3D printed models in the planning process of endovascular procedures with the goal of making a case for using this resource more often. OBJECTIVES/GOALS: To measure the impact of using 3D printed models of patient specific anatomy for pre-procedure planning and as an intra-procedure reference. Impact will be measured by: a. Radiation exposure ; b. Contrast dosage; c. Fluoroscopy time; d. Time to procedural completion; e. ‘Attempts at access,’ when applicable to the procedure METHODS/STUDY POPULATION: Retrospective data will be collected on every patient that received one of prostate artery embolism, transjugular intrahepatic portosystemic shunt placement, or endovascular stent repair in the 3 years prior to the first prospective case. An attempt will be made to create a procedure planning model for every patient that receives one of the three procedures of interest in the 5 months following the first prospective case and those that have a model included in their procedure planning process will be included as part of the experimental group. We anticipate this to not include every patient as there will need to be adequate time between the scheduling of the procedure and the procedure start time to be able to create a 3D model. This will make it impossible to include every patient. Our first prospective case was 11/12/20. RESULTS/ANTICIPATED RESULTS: At the time of submission we have very limited data and cannot confidently make a statement regarding results. We anticipate to measure a reduced time to procedural completion, and as a result, decreased radiation exposure, decreased contrast dosage, and decreased fluoroscopy time in the cases that included a 3D printed model in the planning of the procedures when compared to the procedures that did not include a model. DISCUSSION/SIGNIFICANCE OF FINDINGS: Few hospitals are using 3D printing as a regular tool that physicians can access as a part of their procedure preparation. If we are able to measure a significant impact on the efficiency and safety of procedures in interventional radiology, a much more robust argument can be made for including this technique in procedure planning with regularity.

